# The Effects of Body Acupuncture on Obesity: Anthropometric Parameters, Lipid Profile, and Inflammatory and Immunologic Markers

**DOI:** 10.1100/2012/603539

**Published:** 2012-04-29

**Authors:** Hamid Abdi, Baixiao Zhao, Mahsa Darbandi, Majid Ghayour-Mobarhan, Shima Tavallaie, Amir Ali Rahsepar, Seyyed Mohammad Reza Parizadeh, Mohammad Safariyan, Mohsen Nemati, Maryam Mohammadi, Parisa Abbasi-Parizad, Sara Darbandi, Saeed Akhlaghi, Gordon A. A. Ferns

**Affiliations:** ^1^Departments of Acupuncture Therapy, Beijing University of Chinese Medicine, Beijing 100029, China; ^2^Biochemistry and Nutrition Research Center, Faculty of Medicine, Mashhad University of Medical Sciences (MUMS), Mashhad, Iran; ^3^Cardiovascular Research Center, Faculty of Medicine, Mashhad University of Medical Sciences, Mashhad 9196773117, Iran; ^4^Young Researchers Club, Mashhad Branch, Islamic Azad University, Mashhad 9187147578, Iran; ^5^Institute for Science and Technology in Medicine, Keele University, Thornburrow Drive, Hartshill, Stoke-on-Trent, ST4 7QB, UK

## Abstract

A randomized controlled clinical trial in 196 obese subjects was performed to examine the effectiveness of body acupuncture on body weight loss, lipid profile and immunogenic and inflammatory markers. Subjects received authentic (cases) or sham (controls) acupuncture for 6 weeks in combination with a low-calorie diet. In the following 6 weeks, they received the low-calorie diet alone. Subjects were assessed at the beginning, 6 and 12 weeks later. Heat shock protein (Hsps)-27, 60, 65, 70 antibody titers and high sensitivity C-reactive protein (hs-CRP) levels were also assessed. A significant reduction in measures of adiposity and improvement in lipid profile were observed in both groups, but the levels of anti-Hsp-antibodies decreased in cases only. A reduction in anthropometric and lipid profile in cases were sustained in the second period, however, only changes in lipid profile were observed in the control group. Anti-Hsp-antibodies and hs-CRP levels continued to be reduced in cases but in controls only the reduction in hs-CRP remained. Changes in anthropometric parameters, lipid profile, and anti-Hsp-antibodies were more evident in cases. Body acupuncture in combination with diet restriction was effective in enhancing weight loss and improving dyslipidemia.

## 1. Introduction

Obesity is becoming increasingly common in the general population and is associated with several other conditions such as hypertension, diabetes, dyslipidemia, and cardiovascular disease [1]; these are also increasing in their prevalence. The general principles for the treatment of obesity are to achieve weight loss, to maintain this reduction in body weight following this loss, and to finally reduce the risk factors for obesity. There are several different modalities for treatment of obesity one of which is acupuncture. It would be desirable to control obesity by safe and effective treatment modalities, and among the different methods acupuncture is one of the most popular complementary treatments. Acupuncture is performed by stimulating particular points on the skin called acupoints. Among several methods used for acupoints stimulation [2], needling is one of the most common methods, and stimulation can either be manual or electrical (electroacupuncture). Acupuncture has been used in the treatment of several diseases including obesity [3–5] and also immune-related diseases, such as allergic disorders, autoimmune diseases, and immunodeficiency-syndromes [6–8].

Heat shock proteins (Hsps) are highly conserved proteins expressed by several cell types following exposure to environmental stresses. Over the past 2 decades, there has been an increasing interest in the relationship between Hsps and cardiovascular disease, and particularly whether an autoimmune response may be implicated by formation of anti-Hsp antibodies [9]. In this regard, most of the studies have focused on the relation between cardiovascular disease and Hsp/anti-Hsp-60, -65, -70 while there is recent evidence that supports the role of Hsp/anti-Hsp27 in cardiovascular events (reviewed by Ghayour-Mobarhan et al.) [9]. Wick et al. [10] propose that autoimmunity to Hsps (formation of anti-Hsp antibodies) may contribute to the progression of atherosclerosis [11, 12]. Serum C-reactive protein (CRP) is a feature of systemic inflammation [13] and is positively associated with measures of adiposity such as body mass index (BMI) and waist circumferences (WCs) as demonstrated by 2 large cross-sectional studies [14, 15]. While obesity is related to higher levels of CRP [16], following weight loss reduction in CRP levels has been also reported [17, 18].

There are several studies which have evaluated the effect of acupuncture in the management of obesity. However, most studies have methodological limitations, including small sample size and inadequately controlled study design [19]. To our knowledge, the effect of acupuncture during body weight loss has not been evaluated with respect to inflammatory and immunological markers such as hs-CRP and anti-HSP antibodies. Thus, we aimed to perform a randomized controlled clinical trial in patients suffering from obesity to examine its effectiveness in body weight loss, changing the lipid profile, and inflammatory and immunological markers by body acupuncture.

## 2. Material and Methods

### 2.1. Study Design and Subjects

Two hundred and twenty overweight and obese subjects were recruited from the nutrition clinic, Quem hospital, Mashhad, Iran. In this study, overweight was defined as a BMI of 25 to <30, and a BMI of ≥30 was defined as obesity. They neither had received any other weight control measures nor had any medical and/or drug history within the last 3 months before their participation in the study. Participants were informed about the study both verbally and by written information sheets. Volunteers were given time to discuss the study and were encouraged to ask questions. Those who had exclusion criteria such as diabetes, hypertension, heart disease, endocrine abnormalities, and pregnancy. Finally, 196 subjects were enrolled into the study after checking for the inclusion and exclusion criteria. These subjects were randomized into 2 groups of case and control of body acupuncture (each group had 98 subjects). The subjects were aged between 18 and 55 years and had a body mass index (BMI) between 25 and 45 kg/m^2^. Each patient gave informed written consent to participate in the study, which was approved by the Mashhad University of Medical Science Ethics Committee.

Anthropometric and biochemical assessments were measured before and after treatment. The participants were asked to follow an isocaloric diet (wash-out diet) for 2 weeks before starting the trial to match the two groups for initial diet, and a low-calorie diet for 6 weeks. The low-calorie diet consisted of 1000 kcal deficit per day, less than the individual daily energy expenditure. The resting energy expenditure was calculated using the equation of Harris Benedict [20] and was used to determine the amount of food per day for each participant. The wash-out diet and the 6-week dietary program for each participant were planned by a nutritionist based on the participant's energy expenditure. The diet was prescribed and the participant's compliance was checked every week. 

Hypercholesterolemia was defined using a serum total-cholesterol concentration ≥200 mg/dL or treatment with lipid-lowering drugs, diabetes by fasting plasma glucose level ≥126 mg/dL on ≥2 occasions or treatment with antidiabetic drugs, and hypertension by blood pressures ≥140/90 mmHg on ≥2 occasions or if patients were on treatment with antihypertensive medications [21].

### 2.2. Acupuncture Treatment in Cases

Eight acupuncture points on the abdomen, including Tianshu (ST-25) both sides, Weidao (GB-28) both sides, Zhongwan (REN-12) Shuifen (REN-9) Guanyuan (REN-4) Sanyinjiao (SP-6), and additional Points are Quchi (LI-11) and Fenlong (ST-40) for excess mood (patients with higher energy), and Qihai (REN-6) and Yinlingqau (SP-9) for deficiency mood (patients with lower energy) on both lower legs were selected. In the authentic body-acupuncture group, acupuncture needling with manipulation and use the normal electric lines of the electric acupuncture machine (Ying Lee, KWD 808) to 4 needles on the abdomen, two needles (ST-25) and (GB-28) on each side, was applied for 20 minutes.

In this group, the stainless steel acupuncture needles (3.8 cm long) were inserted to a depth of approximately 2.5 cm after skin sterilization. The needles in the lower legs were manipulated by rotating them back and forth until the subjects had the sensation of *De-Xi*, a term used in acupuncture to describe a feeling of heaviness in the area surrounding the locus of insertion. The needles in the abdomen were applied with electricity. The needles were connected to an electrical stimulator. Electricity was generated as an output of programmed pulse voltage, 30–40 Hz, dense-disperse wave, 390 *μ*S square pulse, and at a maximal tolerable intensity, 500 Ω (12–23 V) (a strong but not painful sensation to the patient). Each acupuncture treatment lasted for 20 minutes. All subjects were asked to receive two treatment sessions per week for a total of 6 weeks. After that all patients in both groups received no acupuncture treatment for next 6 weeks, but were still given advice to keep going on their diet control. All needling was done by a specialized acupuncturist.

In sham acupuncture group, for points on the RN meridian, 0.3 cm laterally to the real location was used, and needling was very superficially as much as possible. Other points are located 0.5 cm up and 0.5 cm laterally to the real location and needling was also very superficially. Disconnected electric lines of the acupuncture machine were applied to 4 needles, each side two needles (ST-25) and (GB-28), for 20 minutes was also used. No major adverse effects were observed in any of patients.

### 2.3. Anthropometric Measurements

For all patients, body weight (BW), BMI, and body fat mass were measured by body composition analyzer BC-418 (TANITA, Japan) according to a standard protocol [22]. Height and body weight were measured with the subjects dressed in light clothing after an overnight fasting. The body weight of each subject was measured with a standard scale to an accuracy of ±0.1 kg, and height was measured to an accuracy of ±0.1 cm. Hip circumference (HC) was measured at the levels of the major trochanters through the pubic symphysis, and waist circumference (WC) was measured midway between the lateral lower rib margin and the iliac crest with the scale to the nearest ±0.1 cm. The body mass index (BMI) was calculated as weight (kg) divided by height squared (m^2^).

### 2.4. Collection of Blood Samples

Blood samples were taken from each patient for analysis after a 12-hour fasting, 3 times during the study (at the beginning, 6 and 12 weeks later). Following venepuncture, blood samples were collected into Vacutainer tubes and centrifuged at 10,000 g for 15 min at 4°C. Hemolyzed samples were excluded from analysis. After separation, aliquots of serum were frozen at −80°C until analysis.

### 2.5. Routine Biochemical Analysis and Serum Anti-HSP-27, 60, 65, 70 Antibody Titers

A full-fasted lipid profile comprising total cholesterol, triglycerides, high-density lipoprotein cholesterol (HDL-C), and low-density lipoprotein-cholesterol (LDL-C) were determined for each subject. Serum lipid and fasting blood sugar (FBS) concentrations were measured enzymatically with the use of commercial kits. hs-CRP was measured by a PEG-enhanced immunoturbidimetry method with an Alycon analyzer (ABBOTT, Chicago, Ill, USA). Serum HSP27, 60, 65, 70 antibody titers were measured using an in-house ELISA assay, as we have explained before [11, 23].

### 2.6. Statistical Analysis

SPSS software (version 16, Chicago, Ill, USA) was used to perform the statistical analysis. Data were checked for normality. Values were expressed as mean ± SEM or, in the case of nonnormally distributed data (like triglyceride), as median and interquartile range. Data that were normally distributed were analyzed using Student's *t*-test. Data found to be nonnormally distributed were analyzed using the nonparametric Mann-Whitney test. For comparison between two related samples, the paired *t*-test (normally distributed data) and the Wilcoxon signed ranks test (non-normally distributed data) were used. For multiple comparisons of parameters Bonferroni corrections were used. A two-sided *P* value of <0.05 was considered statistically significant.

## 3. Results

### 3.1. Demographic Data

**Figure 1:**
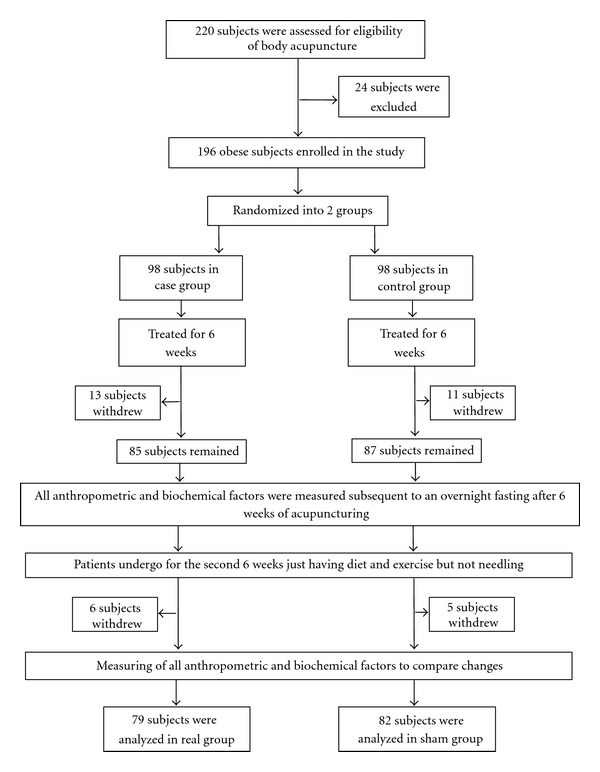
Trial profile and design.

One hundred and ninety-six participants fulfilled the inclusion criteria. Then, they were divided into 2 groups (cases and controls) including 98 subjects. By the end of the study 35 subjects withdrew for personal reasons and 161 participants completed the study (Figure 1). The analysis showed that sex, age, basal levels of anthropometric parameters, lipid profiles, and also levels of anti-Hsp27 antibodies and hs-CRP were not significantly different between case and control groups (*P* > 0.05) at baseline. Clinical and biochemical characteristics of participants have been summarized in Table 1.

### 3.2. Comparison at First 6 Weeks (First Period)

In the authentic acupuncture body group, in the first period of intervention including both acupuncture and low-calorie diet, in authentic acupuncture subjects significant reduction in body weight (*P* < 0.001), body fat percentage (*P* < 0.001), BMI (*P* < 0.001), WC (*P* < 0.001), HC (*P* < 0.001), total cholesterol (*P* < 0.001), triglyceride (*P* < 0.001), HDL-C (*P* < 0.05), and LDL-C (*P* < 0.001) was observed. In the sham body acupuncture group, after 6 weeks of intervention, body weight (*P* < 0.001), body fat percentage (*P* < 0.001), BMI (*P* < 0.001), WC (*P* < 0.001), HC (*P* < 0.001), total cholesterol (*P* < 0.01), HDL-C (*P* < 0.001), and LDL-C (*P* < 0.01) were reduced significantly. Interestingly in the first period of study, all immunological (anti-Hsp antibodies) but not inflammatory (hs-CRP) factors decreased significantly in cases but not in controls (*P* < 0.001), however, just anti-HSP60 antibodies where reduced statistically (*P* < 0.05).  Table 2 summarizes the levels of different parameters.

### 3.3. Comparison at Second 6 Weeks (Second Period)

In the authentic acupuncture group, in the second period of intervention including just low-calorie diet, significant changes were found in body weight (*P* < 0.001), BMI (*P* < 0.01), WC (*P* < 0.001), HC (*P* < 0.001), triglyceride (*P* < 0.01), HDL-C (*P* < 0.001), and LDL-C (*P* < 0.001).

In the control group, significant changes were observed for triglyceride (*P* < 0.01), HDL-C (*P* < 0.001), and LDL-C (*P* < 0.05). The same as the first period of the study, all immunological and inflammatory factors including anti-Hsp antibodies and hs-CRP levels continued their reduction significantly in authentic group (*P* < 0.001), while in controls no statistically changes were seen for these parameters except for hs-CRP (*P* < 0.001) (Table 2).

### 3.4. Comparison the Whole Period of the Study

After 12 weeks of study, in cases of acupuncture body group, significant changes were found in body weight (*P* < 0.001), BMI (*P* < 0.001), WC (*P* < 0.001), HC (*P* < 0.001), total-cholesterol (*P* < 0.001), triglyceride (*P* < 0.001), HDL-C (*P* < 0.001), and LDL-C (*P* < 0.001) (Table 2). In the control group, body weight (*P* < 0.001), body fat percentage (*P* < 0.001), BMI (*P* < 0.001), HC (*P* < 0.001), total cholesterol (*P* < 0.001) and triglycerides (*P* < 0.01), HDL-C (*P* < 0.01), and LDL-C (*P* < 0.001) changed significantly. Overall, immunological and inflammatory factors including anti-Hsp27 antibodies and hs-CRP levels decreased significantly (*P* < 0.001) in the authentic but not control group, except the hs-CRP levels which also significantly reduced in control subjects (*P* < 0.001) (Table 2).

### 3.5. Comparison between Changes in the First and Second Periods of Study

In the acupuncture group, changes in the first (acupuncture and low-calorie diet) versus second (only low-calorie diet) period was significantly different for body weight (*P* < 0.001), BMI (*P* < 0.001), WC (*P* < 0.001), HC (*P* < 0.001), total cholesterol (*P* < 0.001), triglycerides (*P* < 0.01), HDL-C (*P* < 0.001), and hs-CRP (*P* < 0.05). In all above parameters except HDL-C and hs-CRP, the changes were more significant in the first period when compared with second one. In acupuncture control group, body weight (*P* < 0.01), WC (*P* < 0.001), HC (*P* < 0.05), HDL-C (*P* < 0.001), and anti-Hsp27 (*P* < 0.05) were significantly different. All above changes were more significant for the first period of study compared with second period except the HDL-C (Table 3).

### 3.6. Comparison of the Changes in Different Parameters between the Case and Control Groups

For anthropometric parameters, acupuncture was more effective for reducing body weight in all 3 periods of the study (*P* < 0.05), BMI in the first period of the study (*P* < 0.05), WC in all 3 periods of the study (*P* < 0.05), and HC in the first and whole period of the study (*P* < 0.001). For lipid profile, total-cholesterol in the first and whole periods (*P* < 0.05), triglycerides in the first period (*P* < 0.01), and LDL-C in the first and whole period of the study were significantly and more effectively reduced by authentic body acupuncture.

With regards to immunological markers, authentic body acupuncture was more effective as we observed that anti-Hsp27 antibodies were significantly reduced in the all 3 periods of the study when, compared with control acupuncture (*P* < 0.001). The same results were observed for other anti-Hsps (*P* < 0.001). However, the marker of inflammation, hs-CRP was not significantly changed between cases and controls (*P* > 0.05).

## 4. Discussion

**Figure 2:**
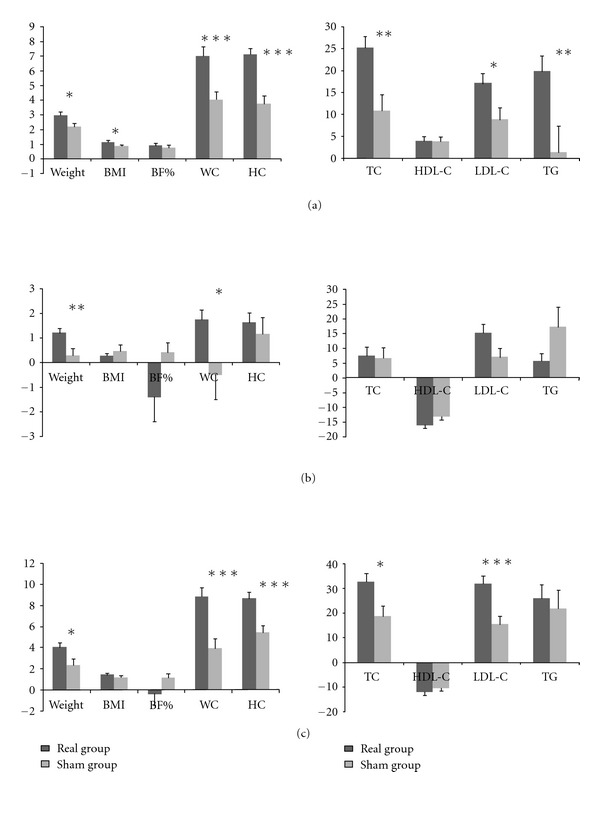
Changes of anthropometric parameters and lipid profile in real and sham groups. BMI: body mass index, BF%: body fat percentage, WC: waist circumferences, HC: hip circumferences, TC: total cholesterol, HDL-C: high density lipoprotein-cholesterol, LDL-C: low density lipoprotein-cholesterol, TG: triglycerides. (a), (b) and (c) means first, second and whole period of the study, respectively, *P < 0.05, **P < 0.01, and ***P < 0.001, respectively.

In the first period of the study, a significant reduction in anthropometric and lipid profile was observed in both cases and controls, but the levels of anti-Hsp antibodies were found to fall in cases but not controls. In the second period which provides evidence for the ability of acupuncture to produce a sustained reduction in anthropometric and lipid profile, whilst in the control group, only changes in lipid profile were sustained. As before, anti-Hsp antibodies and hs-CRP levels continued to fall in cases while no significant changes were observed in controls except hs-CRP. When the whole period of the study was assessed, the reduction in anthropometric, lipid profile and also hs-CRP levels were observed in both cases and controls, but the changes in anti-Hsp antibodies were only statistically significant in the group treated with authentic acupuncture. In addition, the analysis showed that changes in most of the parameters were more significant in the first period of the study when acupuncture were accompanied with diet restriction, compared with second period consisting of diet restriction only. Moreover, changes in anthropometric parameters, lipid profile, and anti-Hsp antibodies were more significant in cases in comparison with the control group. However, the changes in hs-CRP level were not significantly different. Changes in anthropometric and lipid profile in the first, second, and whole period of the study have been summarized in Figure 2.

Consistent with our results, indicating the greater efficacy of body acupuncture therapy for reduction of anthropometric parameters in cases when compared with controls, there are other studies reporting similar findings. Hsu et al. [4] performed a randomized crossover trial in obese women and found that anthropometric parameters changed significantly by electroacupuncture when compared with sit-up exercise. The same results have been found by other studies [3, 5]. However, there are some studies that have reported no significant effect of acupuncture in the treatment of obesity, but it should be noted that these studies were performed by auricular acupuncture therapy [24–26].

It is thought that acupuncture exerts its effects on weight loss through different mechanisms. In terms of traditional medicine, it is believed that acupuncture alters levels of central nervous system by stimulating peripheral nerves at acupoints. Signals are then carried by stimulated nerve resulting in changes in satiety and mood. These mechanisms have been reviewed by Lacey et al. [2]. Acupuncture appears to be able to improve mood by increasing the release of neurotransmitters [27] and suppress appetite by the serotonin and endorphin-induced decreases in stress and depression [28, 29], whereas this effect was not seen by exercise and diet. In addition, it has been shown that application of electroacupuncture at Zusanli (ST-36) and Neiting (ST-44) of the rat caused the increase in the electrical activity of ventral-medical hypothalamus in the obese rat, leading to activation of the satiety center [30]. Waist circumference is related to the subcutaneous fat tissue of the abdomen, and higher effects of body acupuncture in lipolitic activity and enhancing lipid metabolisms could be attributed to the direct effects of body acupuncture in redistribution, lyses of fat tissue [5], and reducing waist circumferences. 

Concerning the effects on lipid profile in both cases and controls, positive changes (increase in levels of HDL-C and decrease in other parameters including triglycerides, total-cholesterol and LDL-C) were observed, however, the changes were significantly different between 2 groups being higher for acupuncture body group, indicating that whilst diet had important effects on the lipid profile but the combination of diet restriction and acupuncture therapy, leads to a further improvement in lipid profile levels. Moreover, Li and Wang [31] have reported significant changes in total and LDL cholesterol in during acupuncture therapy when compared with control subjects. In other study, it was reported that a significant decrease of triglyceride, total cholesterol, LDL-C but no changes in HDL-C in acupuncture group when compared with controls [32]. In several studies, a similar pattern of changes in triglyceride, total-cholesterol, LDL-C, and HDL-C changed has been reported as our study following acupuncture [19, 33, 34], however, 2 of these studies did not find any changes for HDL-C [19, 32], this may be explained by application of different acupoints. It has been suggested that these changes in lipid metabolism may be caused by increase in the serum betaendorphin levels [32].

Reduction in anti-Hsp antibodies in all periods of the study was significant only in authentic group in comparison with controls. Moreover, in the first period of the study when acupuncture combined with diet restriction, this finding was more prominent. To our knowledge, no study has evaluated the effects of acupuncture therapy on these antibodies. However, in a study, it has been reported that dietary constituents are associated with immune response to Hsps in dyslipidemic patients [23]. Our results indicate that acupuncture therapy may have direct effects on immune system and modulates the function of our immunity. As previously noted, these anti-Hsp antibodies have a potential pathologic role in atherogenesis and may progress further inflammatory processes. 

Moreover, as reviewed by Cabyoglu et al. [35], acupuncture is able to increase the levels of encephalin, endomorphin-1, beta endorphin, serotonin, and dopamine and has immunomodulator effects on immune system and lipolitic effects on metabolism. These may be caused by the direct impression of body acupuncture on the adipose tissue of the abdomen. As the results show, body acupuncture was more effective in reduction of waist and hip circumferences, which means the greater losing of adipose tissue of the abdomen, leading to production of less inflammatory markers for the stimulation of the immune system. This role is suggested to be explained by the interaction between the autonomic nervous system (ANS) and the immune functions and the brain as the communicator between 2 systems [36]. By communicating with brain the inflammatory cytokine stimulate neural outflow via the ANS [36], leading to release of acetylcholine via parasympathetic nerve (vagus) endings, resulting to neuroimmune reflex and suppressing the release of inflammatory cytokines [36]. However, it should be noted that this could be a part of the mechanism of how acupuncture works, as we know despite the auricle that vagus nerve is superficial and can be stimulated by acupuncture leading to release of acetylcholine via parasympathetic nerve (vagus) endings, during body acupuncture vagus nerve does not stimulate directly and it should be some other explanatory mechanism.

In addition, reductions in hs-CRP levels were seen in different periods of the study in both groups, interestingly, the analysis showed that changes in hs-CRP levels were not different between cases and control, implying this notion that hs-CRP changed independent of the effects of acupuncture. There are evidences that demonstrate the fact that elevated CRP levels are closely related to obesity [16]. It has been also suggested that adipose tissue releases the proinflammatory cytokines [37], so it is logical that weight loss is associated with decrease in CRP levels. Other studies have found the same result as ours about the reduction in CRP levels following weight loss [17, 18]. Although there are no studies assessing the role of acupuncture on hs-CRP changes during weight loss, but there are some studies which have evaluated the changes in hs-CRP levels in rheumatologic diseases. Similar to our results in patients with rheumatologic problems, some studies reported the inability of acupuncture to reduce the hs-CRP levels [38, 39] and some other not [40].

In conclusion, body acupuncture in combination with diet restriction was found to be effective for weight loss and also reduction of the obesity-associated risks factors, such as dyslipidemia. However, these effects can be achieved by other modalities but due to lack of adverse events and continued effects after the therapy, acupuncture could be used as a proffered or synergic treatment option for obesity control. Moreover, it was found that it has immunomodulatory but not anti-inflammatory effects on immune system by regulation of the levels of anti-Hsp antibodies.

## Figures and Tables

**Table 1:** Comparison between clinical and biochemical characteristics of participants.

Group	Real (*n* = 79)	Sham (*n* = 82)
Age (year)	36.88 ± 0.98	37.41 ± 1.01
Weight (kg)	84.10 ± 1.67	85.02 ± 1.75
Height (cm)	161.08 ± 0.83	160.79 ± 0.92
Body fat (%)	37.00 ± 0.69	37.08 ± 0.79
BMI (Kg/m^2^)	32.30 ± 0.52	32.74 ± 0.59
WC (cm)	102.39 ± 1.28	100.74 ± 1.36
HC (cm)	113.76 ± 1.03	114.98 ± 1.18
WC/HC ratio	0.90 ± 0.1	0.87 ± 0.008
FBS (mg/dL)	84.48 ± 1.46	87.76 ± 2.58
TC (mg/dL)	173.54 ± 4.13	171.96 ± 4.11
TG (mg/dL)	112.48 ± 6.24	123.58 ± 5.8
HDL-C (mg/dL)	42.60 ± 0.99	41.65 ± 1.06
LDL-C (mg/dL)	108.19 ± 3.44	107.46 ± 3.05
DBP (mmHg)	73.33 ± 1.71	75.95 ± 1.67
SBP (mmHg)	107.91 ± 1.92	107.38 ± 1.77
Anti-HSP27 antibody	0.37 (0.32–0.41)	0.35 (0.33–0.41)
Anti-HSP60 antibody	0.74 (0.68–0.86)	0.72 (0.69–0.78)
Anti-HSP65 antibody	0.81 (0.73–0.90)	0.81 (0.78–0.83)
Anti-HSP70 antibody	0.59 (0.55–0.67)	0.61 (0.57–0.66)
hs-CRP (mg/dL)	1.78 (1.15–3.06)	1.79 (0.99–3.39)

BMI: body mass index; WC: waist circumference; HC: hip circumference; FBS: fasting blood sugar; TC: total cholesterol; TG: triglycerides; HDL-C: high-density lipoprotein cholesterol; LDL-C: low-density lipoprotein cholesterol, DBP: diastolic blood pressure, SBP: systolic blood pressure, Anti-Hsp antibody: Anti-heat shock protein, hs-CRP: high-sensitivity C-reactive protein. Values are expressed as mean ± SEM, or median and interquartile range. Chi-square test (for qualitative variable), independent sample *t*-test (for normally distributed data), and Mann-Whitney test (for non-normally distributed data) were used for comparison 2 groups.

**Table 2:** Comparison of clinical and biochemical characteristics of participants of body group.

Variable	Real group			Sham group		
	1st sample	2nd sample	3rd sample	1st sample	2nd sample	3rd sample
Weight (kg)	84.10 ± 1.67	81.23 ± 1.68^*α*^	81.27 ± 2.41^*α*,*β*^	85.02 ± 1.75	82.81 ± 1.81^*α*^	84.96 ± 2.56^*α*^
Body fat (%)	37.00 ± 0.69	36.03 ± 0.7^*α*^	37.91 ± 1.91	37.08 ± 0.79	36.54 ± 0.84^*α*^	36.35 ± 1.01^*α*^
BMI (Kg/m^2^)	32.30 ± 0.52	31.17 ± 0.53^*α*^	30.98 ± 0.62^*α*,*β*^	32.74 ± 0.59	31.87 ± 0.61^*α*^	31.73 ± 0.74^*α*^
WC (cm)	102.39 ± 1.28	95.12 ± 1.2^*α*^	92.90 ± 1.43^*α*,*β*^	100.74 ± 1.36	96.66 ± 1.26^*α*^	97.16 ± 1.31^*α*^
HC (cm)	113.76 ± 1.03	106.59 ± 0.93^*α*^	105.84 ± 1.19^*α*,*β*^	114.98 ± 1.18	111.10 ± 1.15^*α*^	110.40 ± 1.48^*α*^
WC/HC ratio	0.90 ± 0.1	0.89 ± 0.01	0.087 ± 0.01	0.87 ± 0.008	0.87 ± 0.007	0.88 ± 0.008
FBS (mg/dL)	84.48 ± 1.46	87.46 ± 1.33	87.27 ± 3.1	87.76 ± 2.58	89.64 ± 3.40	90.03 ± 3.37
TC (mg/dL)	173.54 ± 4.13	148.18 ± 3.92^*α*^	137.98 ± 3.71^*α*,*β*^	171.96 ± 4.11	162.23 ± 3.72^*α*^	157.15 ± 3.82^*α*^
TG (mg/dL)	112.48 ± 6.24	92.51 ± 4.40^*α*^	87.69 ± 4.55^*α*,*β*^	123.58 ± 5.8	122.97 ± 5.68	108.76 ± 6.37^*α*,*β*^
HDL-C (mg/dL)	42.60 ± 0.99	38.51 ± 0.88^*α*^	54.33 ± 2.96^*α*,*β*^	41.65 ± 1.06	38.16 ± 0.94^*α*^	53.05 ± 3.01^*α*,*β*^
LDL-C (mg/dL)	108.19 ± 3.44	91.34 ± 3.48^*α*^	74.50 ± 3.60^*α*,*β*^	107.46 ± 3.05	99.92 ± 2.89^*α*^	93.27 ± 3.12^*α*,*β*^
Anti-HSP27	0.37 (0.32–0.41)	0.33 (0.29–0.37)^*α*^	0.31 (0.28–0.34)^*α*,*β*^	0.35 (0.33–0.41)	0.36 (0.32–0.39)	0.36 (0.33–0.41)
Anti-HSP60	0.74 (0.68–0.86)	0.73 (0.62–0.80)^*α*^	0.70 (0.62–0.77)^*α*,*β*^	0.72 (0.69–0.78)	0.73 (0.68–0.78)^*α*^	0.73 (0.67–0.78)
Anti-HSP65	0.81 (0.73–0.90)	0.80 (0.73–0.86)^*α*^	0.77 (0.70–0.83)^*α*,*β*^	0.81 (0.78–0.83)	0.80 (0.77–0.84)	0.80 (0.77–0.84)
Anti-HSP70	0.59 (0.55–0.67)	0.56 (0.52–0.64)^*α*^	0.54 (0.50–0.60)^*α*,*β*^	0.61 (0.57–0.66)	0.60 (0.57–0.65)	0.61 (0.56–0.66)
hs-CRP (mg/dL)	1.78 (1.15–3.06)	1.3 (1.3–3.0)	0.8 (0.23–1.4)^*α*,*β*^	1.79 (0.99–3.39)	1.3 (1.3–3.2)	1.10 (0.33–2.00)^*α*,*β*^

R: Real; S: Sham; BMI: Body mass index; WC: waist circumference; HC: Hip circumference; FBS: Fasting blood sugar; TC: total cholesterol; HDL-C: high-density lipoprotein cholesterol; LDL-C: low-density lipoprotein cholesterol; DBP: diastolic blood pressure; SBP: systolic blood pressure. Values are expressed as mean ± SEM, or median and interquartile range. ^*α*^means significant changes in comparison with first sample and ^*β*^means significant changes in comparison with second sample.

**Table 3:** Comparison of changes in clinical and biochemical characteristics of body acupuncture.

Variable	Real group				Sham group				
	1st period	2nd period	Whole period	*P* value 1 v. 2	1st period	2nd period	Whole period	*P* value 1 v. 2	*P* value R. v. S.
									1st *P*: 0.019
Weight (kg)	2.98 ± 0.22	1.22 ± 0.18	4.15 ± 0.37	0.000	2.21 ± 0.23	0.29 ± 0.29	2.40 ± 0.60	0.001	2nd *P*: 0.006
									3rd *P*: 0.012
									1st *P*: 0.632
Body fat (%)	0.90 ± 0.18	−1.39 ± 1.72	−0.38 ± 1.70	0.177	0.77 ± 0.20	0.43 ± 0.40	1.22 ± 0.35	0.507	2nd *P*: 0.326
									3rd *P*: 0.377
									1st *P*: 0.038
BMI (Kg/m^2^)	1.16 ± 0.09	0.29 ± 0.10	1.47 ± 0.14	0.000	0.86 ± 0.10	0.47 ± 0.27	1.24 ± 0.16	0.221	2nd *P*: 0.523
									3rd *P*: 0.294
									1st *P*: 0.000
WC (cm)	7.05 ± 0.60	1.76 ± 0.40	8.93 ± 0.79	0.000	4.07 ± 0.52	−0.50 ± 0.93	3.97 ± 0.92	0.000	2nd *P*: 0.020
									3rd *P*: 0.000
									1st *P*: 0.000
HC (cm)	7.16 ± 0.40	1.63 ± 0.37	8.70 ± 0.56	0.000	3.76 ± 0.56	1.17 ± 0.68	5.49 ± 0.64	0.011	2nd *P*: 0.538
									3rd *P*: 0.000
									1st *P*: 0.926
WC/HC ratio	0.007 ± 0.006	0.003 ± 0.004	0.015 ± 0.008	0.384	0.006 ± 0.005	−0.01 ± 0.008	−0.005 ± 0.008	0.106	2nd *P*: 0.064
									3rd *P*: 0.095
									1st *P*: 0.780
FBS (mg/dL)	−2.70 ± 1.39	−0.70 ± 3.43	−3.61 ± 2.98	0.611	−2.08 ± 1.74	2.83 ± 2.03	0.45 ± 1.58	0.170	2nd *P*: 0.388
									3rd *P*: 0.237
									1st *P*: 0.001
TC (mg/dL)	25.35 ± 2.60	7.52 ± 2.99	32.62 ± 3.59	0.000	10.98 ± 3.58	6.80 ± 3.44	18.51 ± 4.46	0.456	2nd *P*: 0.873
									3rd *P*: 0.014
									1st *P*: 0.002
TG (mg/dL)	19.97 ± 3.55	5.73 ± 2.52	25.97 ± 5.53	0.003	1.48 ± 5.85	17.19 ± 6.84	21.82 ± 7.57	0.530	2nd *P*: 0.200
									3rd *P*: 0.357
									1st *P*: 0.974
HDL-C (mg/dL)	4.02 ± 0.97	−16.07 ± 2.88	−12.12 ± 3.12	0.000	3.97 ± 0.99	−13.15 ± 2.80	−10.64 ± 3.11	0.000	2nd *P*: 0.471
									3rd *P*: 0.739
									1st *P*: 0.017
LDL-C (mg/dL)	17.17 ± 2.23	15.19 ± 2.96	31.85 ± 3.13	0.734	9.03 ± 2.55	7.22 ± 2.80	15.34 ± 3.29	0.581	2nd *P*: 0.054
									3rd *P*: 0.000
									1st *P*: 0.000
Anti-HSP27 Antibody	0.03 (0.01–0.06)	0.02 (0.0–0.04)	0.05 (0.02–0.08)	0.083	0.01 (−0.01–0.02)	−0.01 (−0.02–0.01)	0.0 (−0.01–0.02)	0.028	2nd *P*: 0.000
									3rd *P*: 0.000
									1st *P*: 0.000
Anti-HSP60 Antibody	0.03 (0.02–0.05)	0.02 (0.01–0.05)	0.06 (0.04–0.09)	0.114	0.01 (−0.007–0.02)	0.0 (−0.01–0.02)	0.01 (−0.01–0.02)	0.264	2nd *P*: 0.000
									3rd *P*: 0.000
									1st *P*: 0.003
Anti-HSP65 Antibody	0.02 (−0.01–0.05)	0.03 (0.01–0.05)	0.05 (0.03–0.08)	0.370	0.01 (−0.01–0.02)	0.005 (−0.01–0.017)	0.01 (−0.01–0.02)	0.569	2nd *P*: 0.000
									3rd *P*: 0.000
									1st *P*: 0.000
Anti-HSP70 Antibody	0.02 (0.01–0.04)	0.02 (0.01–0.04)	0.05 (0.03–0.07)	0.936	0.01 (−0.01–0.02)	−0.005 (−0.02–0.01)	0.0 (−0.01–0.02)	0.096	2nd *P*: 0.000
									3rd *P*: 0.000
									1st *P*: 0.790
hs-CRP (mg/dL)	0.0 (−0.63–0.94)	0.90 (0.15–1.7)	1.06 (0.18–1.47)	0.024	0.0 (−0.58–0.75)	0.8 (0.1–1.60)	0.62 (0.13–1.39)	0.226	2nd *P*: 0.691
									3rd *P*: 0.256

R: real; S: sham; BMI: body mass index; WC: waist circumference; HC: hip circumference; FBS: fasting blood sugar; TC: total cholesterol; HDL-C: high-density lipoprotein cholesterol; LDL-C: low-density lipoprotein cholesterol, DBP: diastolic blood pressure, SBP: systolic blood pressure. Values are expressed as mean ± SEM, or median and interquartile range. Paired *t*-test and Wilcoxon signed ranked test were used for comparison between first and second periods for normally and nonnormally distributed data, respectively. Independent sample *t*-test (for normally distributed data) and Mann-Whitney test (for non-normally distributed data) were used for comparison 2 groups.

## Abbreviations

BMI: Body mass index
WC: Waist circumference
HC: Hip circumference
FBS: Fasting blood sugar
TC: Total cholesterol
TG: Triglycerides
HDL-C: High-density lipoprotein cholesterol
LDL-C: Low-density lipoprotein cholesterol
DBP: Diastolic blood pressure
SBP: Systolic blood pressure
Anti-Hsp antibody: Antiheat shock protein and
hs-CRP: High-sensitivity C-reactive protein.
